# Pan‐Cancer Integrative Analyses Reveal the Crosstalk Between the Intratumoral Microbiome, TP53 Mutation and Tumour Microenvironment

**DOI:** 10.1049/syb2.70035

**Published:** 2025-08-28

**Authors:** Baoling Wang, Bo Zhang, Chun Li

**Affiliations:** ^1^ School of Mathematics and Statistics Hainan Normal University Haikou China; ^2^ Geneis Beijing Co. Ltd. Beijing China; ^3^ Precision Medicine Laboratory Mudanjiang Tumor Hospital Mudanjiang China; ^4^ Key Laboratory of Data Science and Intelligence Education of Ministry of Education Hainan Normal University Haikou China; ^5^ School of Elementary Education Hainan Normal University Haikou China

**Keywords:** biology computing, biotechnology, cancer

## Abstract

Accumulating evidence suggests that the TP53 mutation, intratumoral microbiome, and tumour microenvironment (TME) are closely linked to tumourigenesis, yet the biological mechanisms underlying these connections remain unclear. To explore this, we collected multi‐omics data—including genome, transcriptome, and tumour microbiome data—from a wide range of cancer types in The Cancer Genome Atlas (TCGA). Through a pan‐cancer analysis, we identified significant correlations between intratumoral microbiota diversity and TP53 mutation status, particularly in hepatocellular carcinoma (HCC) and endometrial cancer (EC). Despite notable differences in microbiota composition between these two cancer types, we consistently observed that TP53 mutations were associated with reduced alpha‐diversity. Additionally, we found that TP53 mutation status significantly influenced stromal components within the TME, such as a strong correlation between decreased endothelial cell abundance and TP53 mutation. Our integrated approach reveals the complex interplay between TP53 and factors regulating the host TME, offering new insights into cancer progression and potential therapeutic targets for future research.

## Graphical abstract

Pan‐cancer multi‐omics analysis revealed significant correlations between TP53 mutation status, intratumoral microbiome diversity (particularly in HCC and EC) and altered tumour microenvironment (TME) components, such as reduced endothelial cell abundance. Despite compositional differences in microbiota between HCC and EC, TP53 mutations were consistently associated with depleted microbial alpha‐diversity and stromal remodelling in the TME. These findings highlight a multifaceted TP53‐mediated crosstalk between the host TME and intratumoral microbes, offering potential insights for cancer progression and therapeutic strategies.

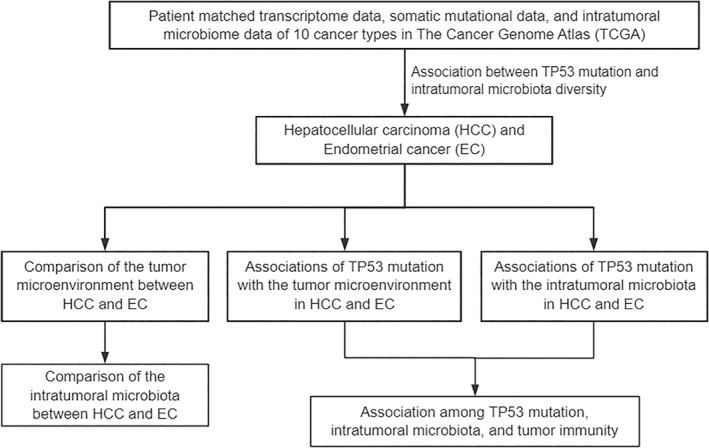

## 1 Introduction

The discovery and presence of the intratumoral microbiome have opened up a novel perspective in cancer research [1, 2]. These intratumoral microbiome, including bacteria, fungi and viruses, are ubiquitously found in various tumour tissues and are closely associated with cancer initiation, progression and prognosis [3, 4]. With the rapid development of high‐throughput sequencing technology, we have gained deeper insights into the diversity and complexity of the intratumoral microbiome [5, 6, 7]. For instance, in colorectal cancer, the enrichment of *Fusobacterium nucleatum* has been confirmed to be tightly linked to cancer progression and poor prognosis. Similarly, in lung cancer, specific bacteria such as *Klebsiella pneumoniae* and *Pseudomonas aeruginosa* have been found to be closely related to tumour growth and metastasis [8, 9, 10]. Intratumoral infection of anaerobic bacteria, such as *Bacteroides*, *Lactobacillus* and *Peptoniphilus*, is correlated with the suppressed antipancreatic ductal adenocarcinoma (PDAC) immunity and poor prognosis [11].

TP53, a crucial tumour suppressor gene, plays a vital role in maintaining genomic stability and preventing cancer development. It is frequently mutated in a wide range of cancers, and these mutations are associated with immune evasion, increased mutation load and alterations in microbiome composition [12, 13]. Recent studies have demonstrated that certain intratumoral microbiome can directly interact with TP53, resulting in its inactivation or further mutation [14]. TP53 mutation may lead to dysbiosis of the gut microbiota, with specific bacteria abnormally enriched in TP53 mutant hosts, potentially exacerbating inflammatory responses [15]. A mutated TP53 gene leads to the loss of p53 protein function, subsequently affecting the normal growth and apoptosis processes of cells [16, 17]. The dysfunctional p53 protein is unable to effectively suppress the proliferation of tumour cells and induce the apoptosis of damaged cells, which in turn promotes the malignant proliferation of cancer cells and enables them to evade apoptosis [18, 19].

The mutation in the TP53 gene not only impacts the biological characteristics of cancer cells but may also promote cancer progression by influencing the tumour microenvironment (TME) [20, 21]. TP53 mutation can lead to the remodelling of TME, thereby influencing tumour cell growth and metastatic potential [22]. James et al. demonstrated that mutated TP53 genes can affect the function of immune cells in the TME, such as T‐cells, B‐cells and natural killer (NK) cells, thereby altering the immune response within the TME and providing favourable conditions for the growth and metastasis of pancreatic cancer cells [23, 24]. This interaction between intratumoral microbiome and TP53 not only deepens our understanding of the complex aetiology of cancer but also opens up new avenues for the development of innovative therapeutic strategies [25]. Tong et al. investigated the potential association among TP53 mutation, the TME, and intratumoral microbiome in lung cancer [26]. However, in other cancer types, this association pattern remains unclear.

**FIGURE 1:**
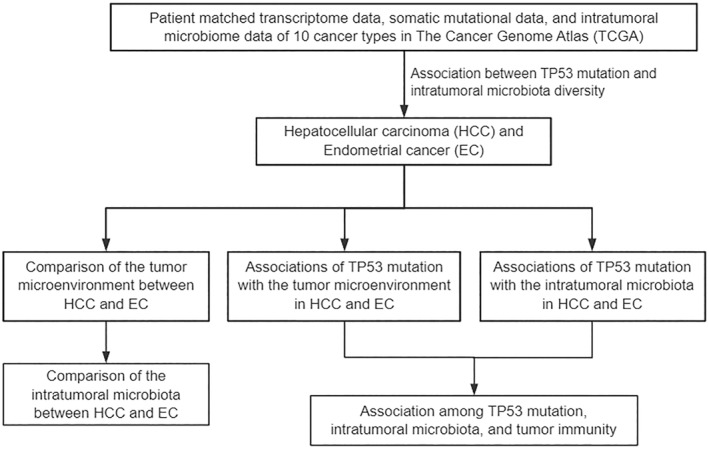
The overall pipeline of this study.

To address this, we performed a systemic correlation between the mutation status of TP53 and intratumoral microbiota profile in a wide range of cancers. The overall pipeline of this study is shown in Figure 1. Consequently, significant associations between the mutation status of TP53 and microbial diversity were observed in hepatocellular carcinoma (HCC) and endometrial cancer (EC). Subsequently, we conducted an integrated multi‐omics profiling covering genome, transcriptome, and microbiome in these two cancers. Complex crosstalk among TP53, specific microbes in tumour and cancer‐associated fibroblasts were detected. Further investigation and validation of the functional mechanisms may facilitate the understanding of the pathogenesis and aetiology of human cancers.

## 2 Materials and Methods

### 2.1 Data Collection

**FIGURE 2:**
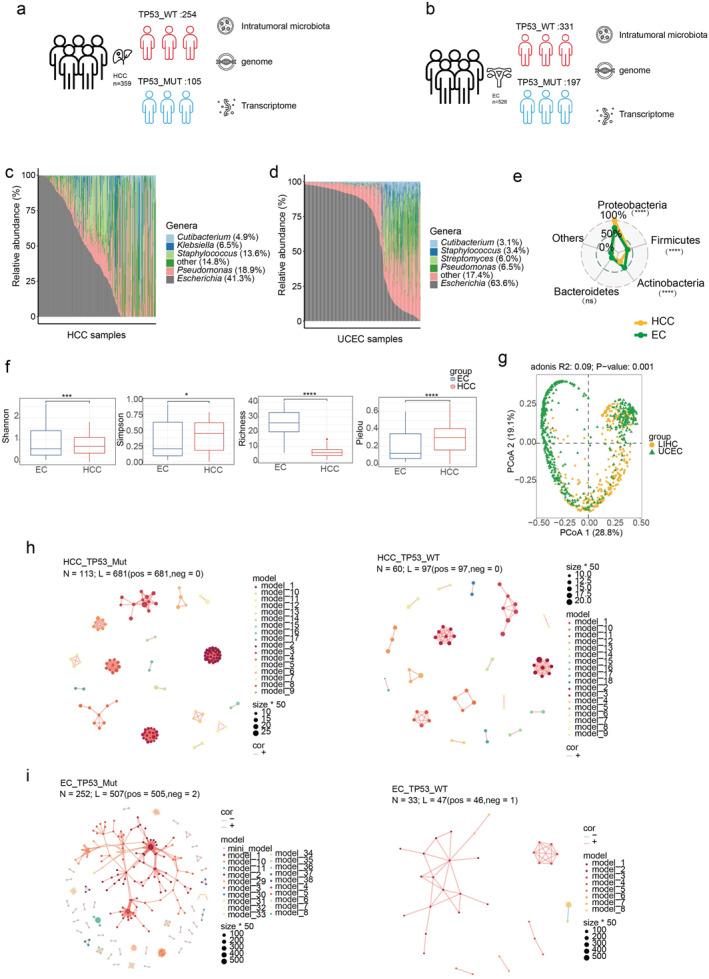
The composition and comparison of the intratumoral microbiota in HCC and EC. (a) The figure legend shows the sample size (359) and grouping (WT: 254, MUT: 105) of HCC samples, as well as the three omics data types (intratumoral microbiome, genome, and transcriptome) investigated in this study. (b) The figure legend shows the sample size (528) and grouping (WT: 331, MUT: 197) of EC samples, as well as the three omics data types (intratumoral microbiome, genome, and transcriptome) investigated in this study. The stacked bar plot shows the five genus‐level bacteria with the highest relative abundance in HCC (c) and EC (d) tumours, whereas the rest are categorised as ‘other’. (e) The radar plot compares the relative abundances of the top four bacterial phyla between HCC and EC samples, with the remaining phyla grouped under ‘Others’. (f) Comparisons in the Shannon index, Simpson index, Richness index and Pielou index between the HCC and EC cancer types. Wilcoxon test, * indicate *p* < 0.05 and **** indicate *p* < 0.0001. (g) The PCoA shows the difference in microbial community composition between the HCC and EC cancer types. The *p* value was generated by PERMANOVA test. The network diagrams show the microbial profiles in the mutated and wild‐type group in HCC (h) and EC (i) tumours. ns, not significant.

According to TP53 gene mutation status, the somatic mutation data from The Cancer Genome Atlas (TCGA) (https://portal.gdc.cancer.gov/) was divided into a wild‐type group (HCC samples, 254 cases; EC samples, 331 cases) and a mutant group (HCC samples, 105 cases; EC samples, 197 cases) (Figure 2a). We have also downloaded gene expression data from TCGA (HCC samples, 359 cases; EC samples, 528 cases). In addition, microbiota data used in this study can be publicly available at TCMbio (https://microbiomex.sdu.edu.cn/). In summary, we collected patient‐matched genome data, transcriptome data, intratumor microbiome data and clinical metadata of 9853 primary tumours and 732 solid normal tissue samples covering 33 types of cancer [27].

### 2.2 Microbial Analysis

Alpha diversity was measured by the Shannon and Simpson index calculated with ‘vegan’ package in R. Alpha diversity including the Shannon, Simpson, Richness, Chao, ACE, Pielou, and Goods_coverage. Shannon index is a comprehensive measure of species richness and evenness. Simpson index indicates the level of diversity, with values ranging from 0 to 1, with values close to 1 indicating an even and abundant distribution of species. Richness directly counts the number of species in a community. ACE index and Chao index are used to estimate the actual number of species in the community. The Pielou evenness index values range from 0 to 1, and close to 1 indicates that the species distribution is highly uniform, which is an important indicator to evaluate the stability of community structure. The Goods_coverage index is used to evaluate the coverage of sequencing data on the true microbial composition of the sample. The higher the value, the more representative the sequencing results are of the true microbial composition of the sample. Beta diversity was measured by Bray‐Curtis dissimilarity index with ‘vegan’ package in R. Linear discriminant analysis Effect Size (LEfSe) was carried out online (http://huttenhower.sph.harvard.edu/galaxy/) to identify microbial biomarkers in TP53 mutation and wild‐type group with a linear discriminant analysis (LDA) score of 2.

### 2.3 Immune Analysis

To quantify the tumour immune infiltration of all samples, six approaches, including TIMER [28], CIBERSORT [29], quanTIseq [30], MCPcounter [31], xCell [32] and EPIC [33], were used to calculate the abundance of various immune cells. In addition, ESTIMATE algorithm was used to estimate the stromal and immune score based on gene expression data [34]. To compare the response of patients with immunotherapy among different cancer, tumour immune dysfunction and exclusion (TIDE) score was calculated online (http://tide.dfci.harvard.edu/) [35].

### 2.4 Prognostic Analysis

In HCC and EC, microbial biomarkers at the genus level were identified using LEfSe between the TP53 mutation and wild‐type groups, respectively. Subsequently, univariate and multivariate Cox regression analyses were performed based on the microbial biomarkers and overall survival (OS) of patients. Thus, patients were divided into two groups based on the results of multivariate Cox regression analysis, indicating the high‐risk and low‐risk groups. Then, log‐rank test was used to perform the statistical test on the OS between the high‐ and low‐risk groups.

### 2.5 Mutation Analysis

The mutation annotation format (MAF) data downloaded directly from the TCGA website can be used directly by maftools to generate a mutation waterfall plot. First, the maftools package in R was used to import the MAF file and perform preliminary analysis. Subsequently, the data was filtered to retain mutations of biological significance. After processing, the filtered mutation data was converted into a format suitable for the ComplexHeatmap package [36], constructing a matrix of samples and mutation types. Finally, the ComplexHeatmap was used to generate the mutation landscape plot, setting the plotting parameters to visualise the distribution and patterns of mutations across different samples.

### 2.6 Statistical Analysis

All statistical analyses in this study were carried out using R software (version 4.3.2). Spearman correlation analysis was used to examine the correlation between intratumoral microbiome and stromal cell. Principal coordinate analysis (PCoA) of Bray‐Curtis distance matrices were performed using the R project, and the results were visualised with the ‘ggplot2’ package in R.

## 3 Results

### 3.1 The TP53 Mutation Is Associated With Intratumoral Microbiota, Especially in HCC and EC

**FIGURE 3:**
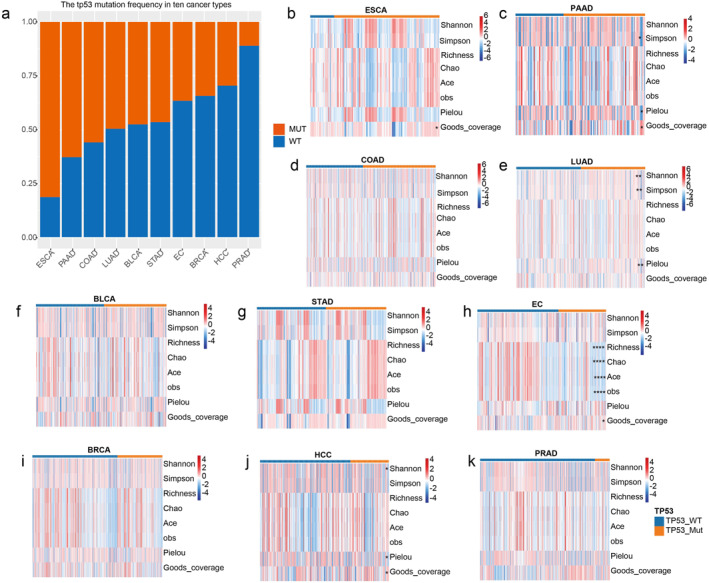
The TP53 mutation is associated with intratumoral microbiota, especially in HCC and EC. (a) TP53 mutation frequency across 10 cancer types. The heatmap displays the comparison of Shannon index, Simpson index, richness index, Chao index, ACE index, observed index, Pielou index and Good's coverage index between TP53 mutation and wild‐type group in ESCA (b), PAAD (c), COAD (d), LUAD (e), BLCA (f), STAD (g), EC (h), BRCA (i), HCC (j) and PRAD (k) tumours.

First, we analysed the frequency of TP53 mutation in 10 types of cancer, including esophageal cancer (ESCA), pancreatic adenocarcinoma (PAAD), colorectal adenocarcinoma (COAD), LUAD, bladder cancer (BLCA), stomach adenocarcinoma (STAD), EC, breast invasive carcinoma (BRCA), HCC and prostate adenocarcinoma (PRAD). There are differences in the frequency of TP53 mutation across these cancer types (Figure 3a). ESCA and PAAD exhibited higher rates of TP53 mutation, whereas PRAD showed a relatively low rate. This indicates that TP53 mutation may play an important role in the occurrence and development of specific cancers, which is consistent with previous research studies [37, 38].

Furthermore, we assessed the correlation between the intratumoral microbiota diversity and TP53 mutation in pan‐cancer (Figure 3b–k). In terms of the alpha diversity, we observed significant associations between alpha diversity and TP53 mutation in PAAD, LUAD, EC and HCC (Figure 3c,e,h and j), supported by at least three alpha diversity indices in each of the three cancer types. Next, we explored the correlation between the intratumoral microbiota composition and TP53 mutation across 10 cancer types (Figure S1a–j). The results suggested substantial associations of the microbiota composition with TP53 mutation in ESCA, HCC and EC. Combining the above results, we detected significant associations of TP53 mutation with both alpha and beta diversity of microbiota composition in HCC and EC. Therefore, next, we focused on these two cancer types to perform subsequent analyses.

### 3.2 The Composition and Comparison of the Intratumoral Microbiota in HCC and EC

A total of 359 HCC samples, including 105 mutant and 254 wild‐type samples (Figure 2a), and 528 EC samples, including 197 mutant and 331 wild‐type samples (Figure 2b), were included in our study. In HCC, the top five abundant genera were *Cutibacterium, Klebsiella, Staphylococcus, Pseudomonas and Escherichia* (Figure 2c). In EC, the top five abundant genera were *Cutibacterium, Staphylococcus, Streptomyces, Pseudomonas and Escherichia* (Figure 2d). We further compared the top four abundant phyla between the two cancer types and found significant differences in the relative abundance of Actinobacteria, Firmicutes and Proteobacteria (Figure 2e). Specifically, Proteobacteria and Firmicutes were significantly higher in HCC than in EC, whereas Actinobacteria and Bacteroidetes were more abundant in EC than in HCC, which was consistent with previous studies [3, 4]. The Shannon, Simpson, and Pielou diversity indices were significantly different between HCC and EC (*p* < 0.0001). Although HCC samples had lower microbial richness compared to EC, significantly higher Shannon, Simpson, and Pielou indices were detected in HCC (Figure 2f).

Furthermore, HCC and EC exhibited significant differences in intratumoral microbial community structure (Figure 2g), suggesting the intertumor heterogeneity. Then, we analysed the microbial co‐abundance network in HCC and EC. The results showed that the TP53 mutation had a significant impact on the intratumoral microbiota. In HCC (Figure 2h), compared to the TP53 mutant group, the wild‐type group exhibited a relatively smaller and more concentrated microbial network, showing fewer positive correlations. In EC (Figure 2i), the mutant group had a more complex network, indicating richer interactions among intratumoral microbiota. Consequently, these results suggest that TP53 mutation may influence the tumour biology by regulating the microbial interactions in tumours.

### 3.3 The Comparison of the TME Between HCC and EC

**FIGURE 4:**
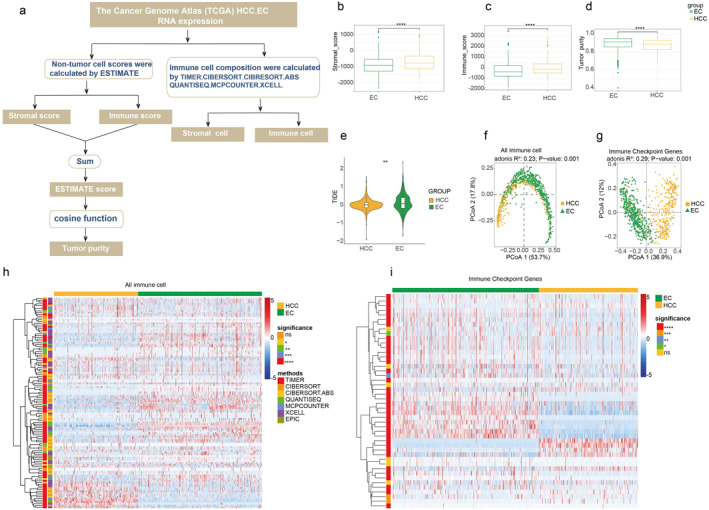
The comparison of the TME between HCC and EC, (a) A process diagram shows how the data on the TME of HCC and EC were obtained. The box plots show the differences in immune scores (b), stromal scores (c) and tumour purity (d) between HCC and EC. (e) The violin plot shows the differences in TIDE scores between HCC and EC. Wilcoxon test, ** indicates *p* < 0.001. The PCoA shows the differences in all immune cells (f) and all immune Checkpoint Genes (g) between HCC and EC. The *p*‐value was generated by the PERMANOVA test. (h) The heatmap shows all immune cells in HCC and EC, indicating the calculation methods for the cells and the significance tests between each pair of cells, using the Wilcoxon test here. * indicate *p* < 0.05 and **** indicate *p* < 0.0001. (i) The heatmap shows immune checkpoint genes in HCC and EC, calculating the significance tests between each pair of genes, using the Wilcoxon test here. ns, not significant.

To compare the TME of two cancer types, we collected relevant data from TCGA and employed appropriate methods for analysis (Figure 4a). We calculated the stromal score and immune score using the ESTIMATE and compared their differences in HCC and EC. The results showed that both the stromal score and immune score in HCC (Figure 4b) were significantly higher than those in EC (Figure 4c). Subsequently, we calculated the tumour purity of these two cancers (Figure 4d) and found that the tumour purity of EC was significantly higher than that of HCC.

We further compared the TIDE scores of HCC and EC (Figure 4e) and found that the TIDE score of EC was significantly higher than that of HCC, implying that the response to immunotherapy of patients with EC may be less favourable. In addition, we observed clear separations in the immune infiltration profile (Figure 4f) and immune checkpoint gene expression (Figure 4g) between these two cancers. We also compared the abundance of various immune cells in HCC and EC (Figure 4h) and found that HCC exhibited more abundant intratumoral immune cell populations. Finally, we compared the expression of immune checkpoint genes in the two cancers (Figure 4i) and found that the expression level of most of immune checkpoint genes was higher in EC, indicating that EC may present stronger immune escape ability. In summary, HCC and EC showed significant differences in the TME profile, immune checkpoint gene expression, and TIDE score. These findings provide important insights for further exploring the biological characteristics and therapeutic strategies of these two cancers.

### 3.4 The Mutation Status of TP53 Is Related to the TME

**FIGURE 5:**
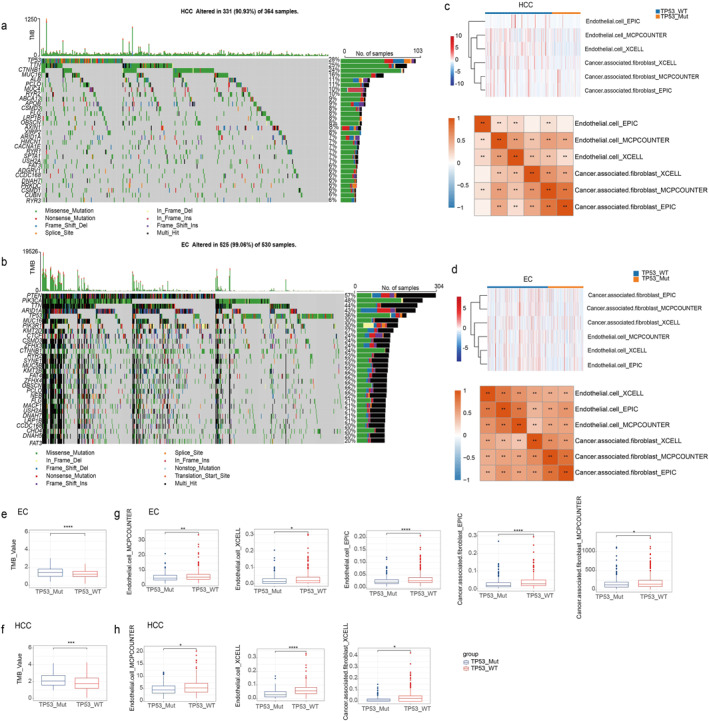
The mutation status of TP53 is related to the TME. The mutation landscape shows the types of mutations and their proportions in HCC (a) and EC (b) tumours. The heatmap shows the differences in stromal cells between the TP53 mutated and wild‐type group in HCC (c) and EC (d) tumours. The correlation heatmap below the heatmap showing the correlations between the abundances of stromal cells calculated by different methods. The box plot shows the differences in TMB values between the TP53 mutated and wild‐type group in HCC (e) and EC (f) tumours. (g) The box plot shows five types of stromal cells that have significant differences between the TP53 mutated and wild‐type group in EC tumours. (h) The box plot shows three types of stromal cells that have significant differences between the TP53 mutated and wild‐type group in HCC tumours. Wilcoxon test, * indicate *p* < 0.05, ** indicate *p* < 0.01, ***indicate *p* < 0.001,**** indicate *p* < 0.0001.

Next, we identified the 30 genes with the highest frequency of mutation in HCC (Figure 5a) and EC (Figure 5b), with mutation frequencies ranging from 6% to 28% in HCC and 20%–57% in EC. In HCC, TP53 emerged as the most commonly mutated gene, with a mutation frequency of up to 28%. Additionally, genes related to the Wnt pathway, such as CTNNB1 and AXIN1, frequently exhibited mutations, indicating that TP53 inactivation and Wnt pathway aberrations are significant mechanisms in HCC development. In EC, the TP53 mutation frequency reached 36%, and different genes displayed distinct mutation patterns, highlighting the diverse mutation mechanisms present in this cancer type. We analysed the differences in tumour mutational burden (TMB) between the mutation and wild‐type group in HCC (Figure 5e) and EC (Figure 5f). The results showed that the mutation groups had significantly higher TMB values compared to the wild‐type group.

Next, we analysed the differences in the abundance of six stromal cell types between the mutation and wild‐type group in HCC (Figure 5c) and EC (Figure 5d). The results showed significant differences, indicating that TP53 mutation may lead to a more mesenchymal and angiogenesis‐suppressed TME. Specifically, in HCC, the abundances of endothelial cells and cancer‐associated fibroblasts (CAFs) were significantly lower in the mutation group compared to the wild‐type group (Figure 5h), and similar results were detected in EC (Figure 5g). It is worth noting that we found that the abundance of stromal cells calculated by different methods showed a significant positive correlation, indicating the consistency of different methods in evaluating the TME.

In summary, TP53 gene mutation may increase the TMB and lead to a TME shift towards suppressed angiogenesis and increased mesenchymal features, thereby influencing the progression and treatment outcomes of HCC and EC. This provides important clues for further investigating the role of TP53 in tumour development and immune regulation.

### 3.5 The Relationship Between TP53 Mutation and Intratumoral Microbiota

**FIGURE 6:**
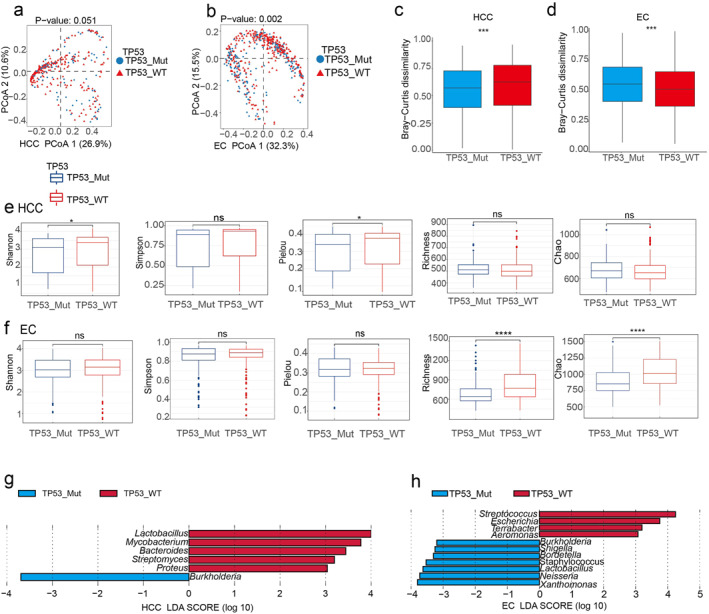
The relationship between TP53 mutation and intratumoral microbiota. The PCoA shows the differences in microbial communities based on TP53 mutation status in HCC (a) and EC (b) tumours. A *p*‐value is generated through the PERMANOVA test. This box plot shows the *β* diversity based on Bray distance in the TP53 mutation group and wild‐type group in HCC (c) and EC (d) tumours, with the *p*‐value based on the Kruskal–Wallis statistical test. The box plot shows the comparison of the Shannon index, Simpson index, Pielou index, richness index, and Chao index between the TP53 mutant and wild‐type group in HCC (e) and EC (f) tumours. In HCC (g) and EC (h) tumours, the enriched microorganisms at the genus level in the mutant and wild‐type group were identified through LEfSe analysis, with an LDA value of 3.

To further explore the impact of TP53 mutation on the intratumoral microbiota, we divided the samples into TP53 mutation and wild‐type group and conducted a PCoA analysis on HCC (Figure 6a) and EC (Figure 6b). It is interesting that there are significant differences in intratumoral microbiota between the TP53 mutant group and wild‐type group. Further analysis of the Bray–Curtis dissimilarity revealed that in HCC, the dissimilarity was lower in the TP53 mutant group compared to the wild‐type group (Figure 6c), whereas in EC, the dissimilarity was higher in the TP53 mutant group compared to the wild‐type group (Figure 6d). The analysis of intratumoral microbiota alpha diversity showed that in HCC, the TP53 mutant group had significantly lower Shannon and Simpson indices compared to the wild‐type group (Figure 6e). In EC, the TP53 mutant group had significantly lower Richness and Chao indices compared to the wild‐type group, also suggesting a reduction in community diversity (Figure 6f).

To assess the specific influence of TP53 mutation on the intratumoral microbial communities in HCC and EC patients, next, LEfSe was used to identify the differential abundant microbes. In HCC, the genus *Burkholderia* was found to be more abundant in the TP53 mutant group, while the wild‐type group showed higher abundance of *Lactobacillus, Mycobacterium, Bacteroides, Streptomyces,* and *Proteus* (Figure 6i). In EC, the TP53 mutant group was enriched for *Xanthomonas, Neisseria, Lactobacillus, Staphylococcus, Bordetella,* and *Shigella*, while in the TP53 wild‐type group, the relative abundance of *Streptococcus, Escherichia, Terrabacter,* and *Aeromonas* were elevated (Figure 6h). In summary, the changes in the microbial community may participate in the development and progression of the tumours, and TP53 gene mutation is one of the important regulatory factors in this process. Further in‐depth investigation of the complex mechanisms between TP53 gene and tumour microecology is needed to provide new targets and strategies for cancer prevention and treatment.

### 3.6 The Relationship Between Intratumoral Microbiota, TP53 Mutation, and TME

**FIGURE 7:**
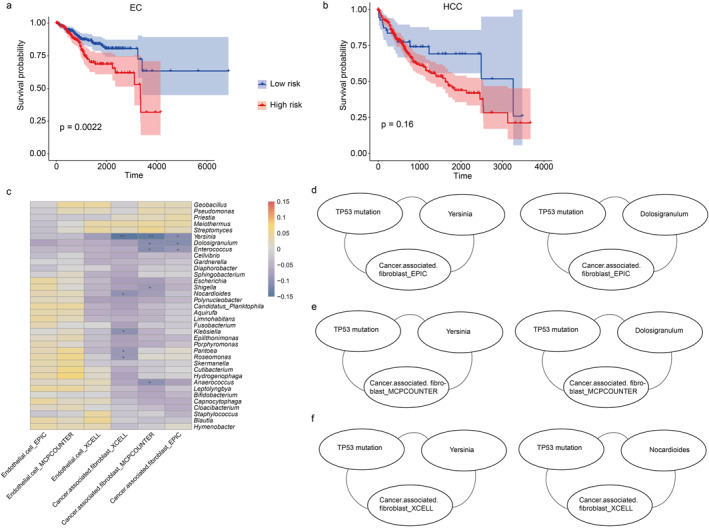
The relationship between intratumoral microbiota, TP53 mutation, and TME. Using the intratumoral microbiota identified from LEfSe analysis with an LDA value of 2 as input, we performed Cox analysis and derived survival curves for EC (a) and HCC (b), based on the median risk score as the cutoff point to stratify patients into high‐risk and low‐risk groups. The relationship between intratumoral microbiota and stromal cells in EC (c), with significant relationships marked by an asterisk (*). The relationship between cancer.associated fibroblasts _EPIC (d), cancer.associated fibroblasts _MCPCOUNTER (e), cancer.associated fibroblasts _XCELL (f) intratumoral microorganisms, and TP53 mutation.

Based on the microbial biomarkers identified by LEfSe, we conducted Cox proportional hazards analysis. Subsequently, we generated survival curves for patients with EC and HCC, utilising the median risk score as a cut‐off to stratify individuals into high‐risk and low‐risk subgroups. The outcomes revealed statistically significant disparities in OS between the two risk groups in EC (Figure 7a), highlighting the prognostic value of these intratumoral microbiota signatures. However, we did not observe significant difference in OS between the two groups in HCC (Figure 7b). Thus, we focused on EC to perform further analyses.

We found that mutation status of TP53 was related to the diversity of intratumoral microbiota and the stromal cells in the TME; therefore, we performed a correlation analysis between intratumoral microbiota and stromal cells using Spearman's correlation coefficient, and the results revealed significant associations between specific intratumoral microbes and stromal cells (Figure 7c). Furthermore, we found that the intratumoral microbes that had significant correlations with cancer‐associated fibroblasts included *Yersinia*, *Nocardioides*, *Klebsiella*, *Pantoea* and *Roseomonas*. Indicating that these intratumoral microbes might have played important roles in regulating the TME. Additionally, we observed some potential connections between TP53 mutation and the two factors of intratumoral microbes and the TME (Figure 7d–f).

## 4 Discussion

Recent studies showed that the occurrence and progression of cancer were associated with TP53 mutation [15, 39]. Intratumoral microbiota has been reported to influence and regulate the development and progression of cancer [40, 41]. Additionally, there was a relationship between the TME and cancer [42, 43]. However, research exploring the interplay among TP53 mutation, intratumoral microbiota, and the TME in the context of cancer remains limited. This article investigated the relationships among these three aspects in the setting of cancer.

Firstly, the high frequency of TP53 mutation in both HCC and EC, along with their impact on microbial communities, suggests that TP53, as an important tumour suppressor gene, may play a critical role in tumourigenesis and progression. We observed that the microbial diversity in the TP53 mutant group was significantly lower than that in the wild‐type group for both cancers (Figure 6e,f). This phenomenon has been recorded in various cancer types [44, 45], indicating that TP53 mutation may lead to an imbalance in microbial communities, which could affect tumour growth and response capabilities. Particularly in HCC, lower Shannon and Simpson diversity indices reflect a significant impact on the richness and evenness of the microbial community, potentially indicating the loss of certain key microorganisms [46], thereby affecting the ecological balance of the TME. A large number of studies reported that gut microbes could promote cancer development by regulating P53 expression, such as *Escherichia coli* and *Helicobacter pylori* [47, 48]. However, the complex relationship between the microbial community in the TME and TP53 gene mutations remains poorly understood. Given the critical role of TP53 mutation in prognosis and immune infiltration of multiple cancers [49], uncovering the relationship between TP53 mutations and the composition of the tumour microenvironment is of great significance.

We observed substantial variation in intratumoral microbiome between HCC and EC, suggesting significant tumour heterogeneity. Previous studies also reported that tumours harbour specific intratumoral microbiota profiles [50]. Specific, our study showed that the richness of microbial community in EC was significantly higher than that of HCC. This may be due to the fact that the endometrium is located within the female reproductive tract and is directly connected to the vagina and cervix, forming a continuous microbial niche. The vaginal microbiota itself is highly diverse, and the microorganisms in it can ascend to the endometrium through the cervical canal. The liver microbes are mainly derived from the translocation of intestinal flora, and their species composition is strictly restricted by the intestinal microbiota (such as Firmicutes and Bacteroidetes). Despite the diversity of gut microbiota, there are only a limited number of bacteria that can break through the intestinal mucosal barrier and colonise the liver.

Strategies to therapeutically target the TME have emerged as a promising approach for cancer treatment in recent years due to the critical roles of the TME in regulating tumour progression and modulating response to standard‐of‐care therapies [51]. Regarding the TME, our analysis showed that the level of immune cell infiltration in HCC was significantly higher than in EC (Figure 4c), indicating a potentially stronger immune response in the HCC TME. The lower tumour purity observed in HCC may stem from its abundant stromal components and the extent of immune cell infiltration [52]. Lower tumour purity might confer a greater advantage for immune evasion in HCC, which could significantly impact the effectiveness of immunotherapy. Studies indicate that the types and abundances of immune cells in the TME significantly affect responses to immunotherapy, thus understanding the differences between HCC and EC in this regard may provide new insights for personalised treatment.

We also showed a close relationship between TP53 mutation and the TME characteristics. In both HCC and EC, patients with TP53 mutation exhibited depleted abundance of stromal cells, such as endothelial cells and CAFs. Consistently, studies have shown that TP53 mutant lung adenocarcinoma tumours have a significantly lower proportion of endothelial cells than wild‐type tumours, resulting in restricted angiogenesis [53]. CAFs secrete cytokines such as IL‐6 and TGF‐β to inhibit the infiltration of CD8^+^ T cells and form an immunosuppressive microenvironment [54]. We observed significantly higher TMB in TP53 mutant tumours which indicates a promising response to immune checkpoint inhibitors (ICIs). Thus, a potential explanation may be due to reduced CAFs in TP53 mutant tumours.

In the study of intratumoral microbiota, we also observed significant differences in the intratumoral microbiota structure between the TP53 mutant and wild‐type group. These differences not only reflect changes in microbial diversity but may also impact immune responses within the TME. For example, in HCC, the abundance of *Burkholderia* increased in the TP53 mutant group, while the wild‐type group was dominated by beneficial bacteria such as *Lactobacillus* (Figure 6g), which may affect the tumour's ability to evade immune responses [55]. A diverse intratumoral microbiome plays a pivotal role in shaping immunotherapy response [56, 57]. Greater microbial diversity fosters a more robust immune infiltration, including crucial anti‐tumour cells. This diversity influences immune activation through metabolites and antigen mimicry. Strategies to enhance microbial diversity, such as faecal microbiota transplantation, can reshape the tumour immune landscape, boosting therapy success. It is noted that our results are limited to correlations and cannot establish a causal relationship between the microorganisms and TP53 mutations. To address this, in vitro and in vivo experiments combined with multi‐omics sequencing data are warranted.

Finally, our research study reveals the complex interactions among TP53 mutation, intratumoral microbiota and the TME. These findings provide important clues for understanding the ecological characteristics of the TME and point the way for future research. Future studies should further investigate how TP53 mutation specifically affect microbial communities and their roles in tumour development. These goals cannot be achieved without the application of artificial intelligence algorithms and single‐cell sequencing technology [58, 59]. AI algorithms can analyse single‐cell multi‐omics data to map TP53 mutation landscapes at clonal resolution, identifying subpopulations with distinct mutation patterns (e.g., biallelic/multi‐hit mutations). By integrating single‐cell transcriptomics and microbial metagenomics, machine learning models can predict TP53‐mutant‐driven inflammatory microenvironments and microbial dysbiosis [60]. Deep learning frameworks can then simulate tumour–microbe–immune interactions, revealing how TP53 mutations alter microbial composition to promote tumour progression via chronic inflammation or metabolic reprogramming.

TP53‐microbiome signatures offer actionable insights for precision oncology. By linking TP53 mutations to distinct microbial profiles, patients could be stratified into subgroups with differential immunotherapy responsiveness, enabling tailored treatment selection. These interactions also suggest microbiota‐targeted interventions: for instance, restoring antitumour microbial communities via probiotics or FMT in TP53‐mutant patients, or selectively depleting immunosuppressive taxa with antibiotics. Prospective clinical trials integrating TP53‐microbiome profiling with such strategies could refine therapeutic approaches, optimise response rates and ultimately improve survival outcomes in cancer patients.

Our study also had several limitations. Firstly, because of the retrospective nature of the study, we did not assess the potential influence of clinical variables as confounders on findings, such as age, sex, and tumour stage. In the future, more prospective designs should be used to explore the association between tumour microbiota, gene mutations and the tumour microenvironment. Additionally, while we observed significant differences in microbial communities between TP53 mutant and wild‐type group in HCC and EC, the functional implications of these differences remain largely unknown. Further studies are required to investigate the potential roles of these microbial species in tumourigenesis, progression, and response to therapy. Finally, our findings are based on observational data, and causality cannot be inferred. Future functional research should be incorporated into co‐culture systems or gnotobiotic mouse models. For example, TP53 mutant and wild‐type HCC cells can be co cultured with endothelial cells or immune cells, and *Burkholderia* or its metabolites can be introduced to observe the effects on cell behaviour, such as proliferation, migration and secretion of inflammatory factors in the co culture system.

In summary, our study highlights the significance of the mutation status of TP53 in HCC and EC, revealing the complex relationships between intratumoral microbiota and the TME. Our findings provide novel insights into the biological characteristics of tumours, and prognostic evaluations, holding significant promise for advancing personalised treatment strategies.

## Author Contributions

**Baoling Wang:** formal analysis, visualization, writing – original draft, writing – review and editing. **Bo Zhang:** formal analysis, investigation, visualization, writing – original draft. **Chun Li:** conceptualization, funding acquisition, project administration, writing – review and editing.

## Conflicts of Interest

The authors declare no conflicts of interest.

## Supporting information


**Figure S1:** PCoA plots of ten cancer types. PCoA showing the differences in microbial communities based on TP53 mutation status in (a) ESCA, (b) PAAD, (c) COAD, (d) LUAD, (e) BLCA, (f) STAD, (g) PRAD, (h) BRCA, (i) HCC, and (j) EC tumors. A *p*‐value is generated through the PERMANOVA test.

## Data Availability

The data that support the findings of this study are available from https://portal.gdc.cancer.gov/ and https://microbiomex.sdu.edu.cn/.
