# Supplementary material for: Pan‐Cancer Integrative Analyses Reveal the Crosstalk Between the Intratumoral Microbiome, TP53 Mutation and Tumour Microenvironment
Source: IET Syst Biol. 2025 Aug 28;19(1):e70035. doi: 10.1049/syb2.70035 (PMC12393667; doi:10.1049/syb2.70035)
Supplement: Supplementary file 1 — Figure S1: PCoA plots of ten cancer types. PCoA showing the differences in microbial communities based on TP53 mutation status in (a) ESCA, (b) PAAD, (c) COAD, (d) LUAD, (e) BLCA, (f) STAD, (g) PRAD, (h) BRCA, (i) HCC, and (j) EC tumors. A p‐value is generated through the PERMANOVA test. [file SYB2-19-e70035-s001.docx]

**Pan-cancer integrative analyses reveal the crosstalk between the intratumoral microbiome, TP53 mutation and tumor microenvironment**

Baoling Wang1,2#, Bo Zhang3# , Chun Li1*

1School of Mathematics and Statistics, Hainan Normal University, Haikou, 571158, China

2Geneis Beijing Co., Ltd., Beijing, 100102, China

3Precision Medicine laboratory, Mudanjiang Tumor Hospital, Mudanjiang,157009,China

#The authors contributed to this work equally

*Correspondence: Chun Li, E-mail: lichunlizhg@163.com

The submitting author's email address is: [1742636892@qq.com](https://mailto:1742636892@qq.com/)


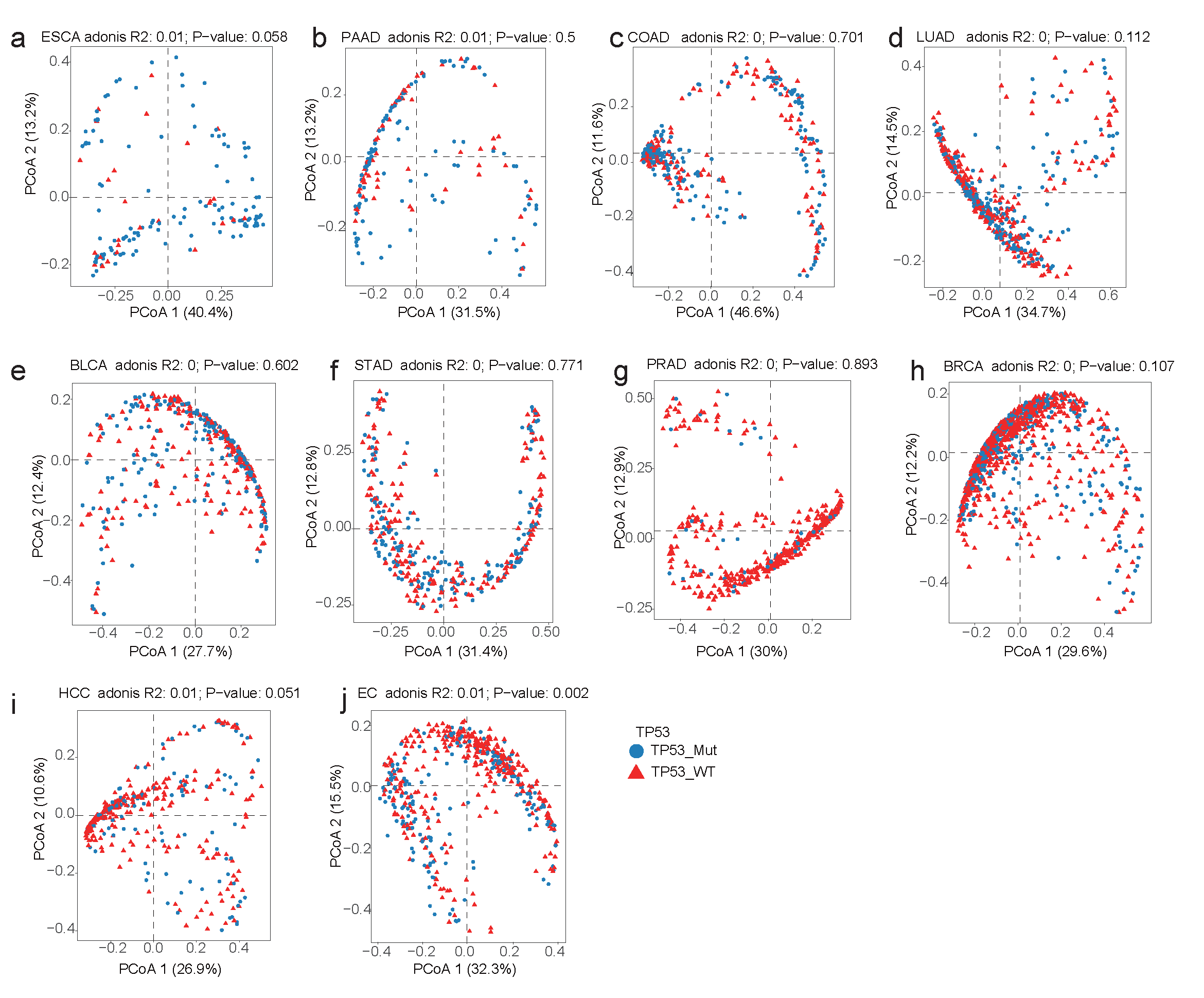


**Figure S1. PCoA plots of ten cancer types.**

PCoA showing the differences in microbial communities based on TP53 mutation status in (a) ESCA, (b) PAAD, (c) COAD, (d) LUAD, (e) BLCA, (f) STAD, (g) PRAD, (h) BRCA, (i) HCC, and (j) EC tumors. A p-value is generated through the PERMANOVA test.
